# Effect of context exposure after fear learning on memory generalization in mice

**DOI:** 10.1186/s13041-015-0184-0

**Published:** 2016-01-08

**Authors:** Ayano Fujinaka, Ruoshi Li, Masanobu Hayashi, Deependra Kumar, Gopakumar Changarathil, Keisuke Naito, Kousuke Miki, Taihei Nishiyama, Michael Lazarus, Takeshi Sakurai, Nohjin Kee, Satomi Nakajima, Szu-Han Wang, Masanori Sakaguchi

**Affiliations:** International Institute for Integrative Sleep Medicine (WPI-IIIS), University of Tsukuba, Tsukuba, Japan; Department of Molecular Neuroscience and Integrative Physiology, Faculty of Medicine, Kanazawa University, Ishikawa, Japan; University of Toronto, Toronto, Canada; Department of Adult Mental Health, National Center of Neurology and Psychiatry, National Institute of Mental Health, Tokyo, Japan; Centre for Clinical Brain Sciences, University of Edinburgh, Edinburgh, UK

**Keywords:** Fear memory, Generalization

## Abstract

**Background:**

The conditions under which memory generalization occurs are not well understood. Although it is believed that fear memory generalization is gradually established after learning, it is not clear whether experiences soon after learning affect generalization.

**Results:**

Using a contextual fear conditioning paradigm in mice, we found that fear memory generalization occurred when mice were exposed to a familiar, unconditioned context soon after fear learning.

**Conclusions:**

Our results suggest that the familiarity of contexts and the timing of their exposure influences memory generalization, which increases our understanding of the mechanisms of generalization.

**Electronic supplementary material:**

The online version of this article (doi:10.1186/s13041-015-0184-0) contains supplementary material, which is available to authorized users.

## Background

One definition of memory generalization is the occurrence of learned responses in circumstances that differ from those prevailing during memory acquisition [1]. Although memory generalization can be beneficial if it is balanced with discrimination, in patients with post-traumatic stress disorder, for example, over-generalization can lead to more frequent reminders of the traumatic events, which can hamper quality of life [2]. Therefore, understanding the conditions under which memory generalization occurs would not only advance memory research but could also contribute to the development of better treatments for patients who show over-generalization [3, 4].

The contextual fear conditioning (CFC) paradigm in rodents has been extensively used to investigate the mechanisms of memory generalization. In a standard CFC protocol, a rodent associates a specific context with an electric foot shock and thus shows a freezing response when it is exposed to the same context in which it received the foot shock. If a freezing response also occurs in contexts that were not associated with shock, this can indicate that the animal generalized (i.e., failed to discriminate) among contexts [5–11].

Some brain regions that are involved in memory generalization have been identified [5–8, 12–17]. In addition, several predisposing factors occurring both before and after fear learning are known to contribute to generalization [10, 18, 19]. However, the conditions that influence memory generalization shortly after fear learning remain to be clarified. Here, we provide evidence that re-experiencing a familiar context soon but not long after fear learning increases memory generalization.

## Results

### Context exposure soon after training induces generalization of contextual fear memory

**Fig. 1:**
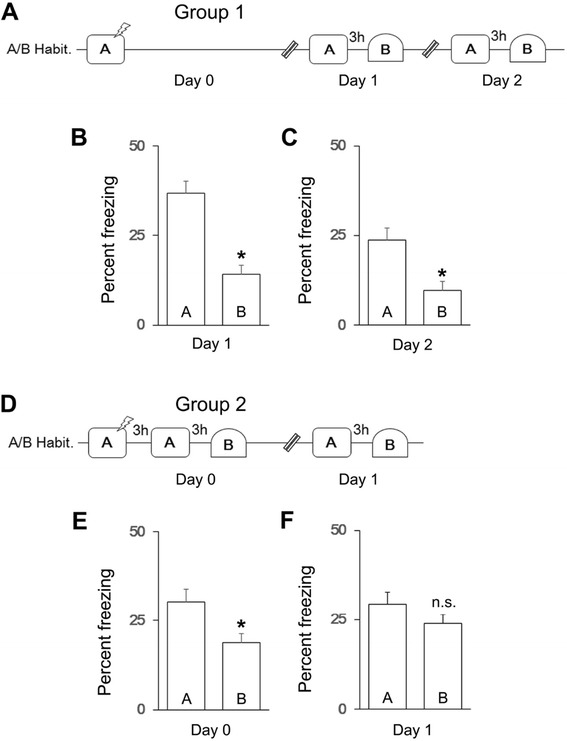
Fear generalization when mice were exposed to contexts soon after training. **a** Experimental timeline for mice in Group 1. Freezing in each context on (**b**) day 1 and (**c**) day 2 after training**. d** Experimental timeline for mice in Group 2. Freezing in each context on (**e**) day 0 and (**f**) day 1 after training. A/B Habit. indicates habituation to contexts A and B before training. The double diagonal line in the timelines indicates a change in day, and the lightning bolt indicates the foot shock. Post-hoc analyses results: *p < 0.05, n.s. = not significant

First, we examined whether mice in Group 1 (Fig. 1a, *n* = 15) discriminate between contexts A and B (Additional file 1: Figure S1) 24 h after training in context A. We found that mice exhibited an increase in freezing in context A after training (A_habituation_ < A_day1_, paired *t*-test, *t*(14) = 9.3, *p* < 0.0001, data not shown). Mice also showed greater freezing in context A than in context B on both day 1 and day 2 (Fig. 1b–c, context × day ANOVA, main effect of context, *F*(1,14) = 64.3, *p* < 0.01; post-hoc analysis, A_day1_ > B_day1_, paired *t*-test, *t*(14) = 7.5, *p* < 0.001, A_day2_ > B_day2_, paired *t*-test, *t*(14) = 6.6, *p* < 0.001), although some degree of forgetting was observed across days (context × day interaction, *F*(1,14) = 11.93, *p* < 0.01, main effect of day, *F*(1,14) = 23.5, *p* < 0.005; post-hoc analysis, A_day1_ > A_day2_, paired *t*-test, *t*(14) = 9.3, *p* < 0.001). These results suggest that context exposure 24 h after training does not induce memory generalization.

Next, we examined whether exposure to contexts A and B at an earlier time point after training induces generalization in mice in Group 2 (Fig. 1d, *n* = 30). We found that mice exhibited an increase in freezing in context A after training (A_habituation_ < A_day0_, paired *t*-test, *t*(29) = 9.9, *p* < 0.0001, data not shown). Although mice exhibited higher levels of freezing in context A than in context B on both day 0 and day 1 (Fig. 1e–f, context × day ANOVA, main effect of context, *F*(1,29) = 16.7, *p* < 0.01), the pattern of freezing exhibited by mice in Group 2 on day 1 differed from that exhibited by mice in Group 1 on day 2 (compare Fig. 1f to c, group × context ANOVA, group × context interaction, *F*(1,43) = 4.1, *p* < 0.05). Specifically, whereas mice in Group 1 discriminated between contexts on day 2, mice in Group 2 showed generalization on day 1 (Fig. 1f, post-hoc analysis, A_day1_ ≅ B_day1_, paired *t*-test, *t*(29) = 2.0, *p* > 0.05), indicating that the timing of context exposure after training affects generalization.

Furthermore, we found that generalization between contexts depends on habituation to the contexts before training, as the pattern of freezing on day 1 differed between mice in Group 2 and mice in Group 8 (compare Fig. 1f and Additional file 1: Figure S2C, group × context ANOVA, group × context interaction, *F*(1,38) = 7.0, *p* < 0.05), which did not receive habituation (Additional file 1: Figure S2A-C, context × day ANOVA, main effect of context, *F*(1,9) = 29.2, *p* < 0.01, main effect of day, *F*(1,9) = 8.9, *p* < 0.05; post-hoc analysis, A_day0_ > B_day0_, paired *t*-test, *t*(9) = 2.3, *p* < 0.05, A_day1_ > B_day1_, paired *t*-test, *t*(9) = 3.3, *p* < 0.01). Together, these results suggest that memory generalization depends on the familiarity and timing of context exposure after training.

### Exposure to a familiar context soon after training induces generalization of contextual fear memory

**Fig. 2:**
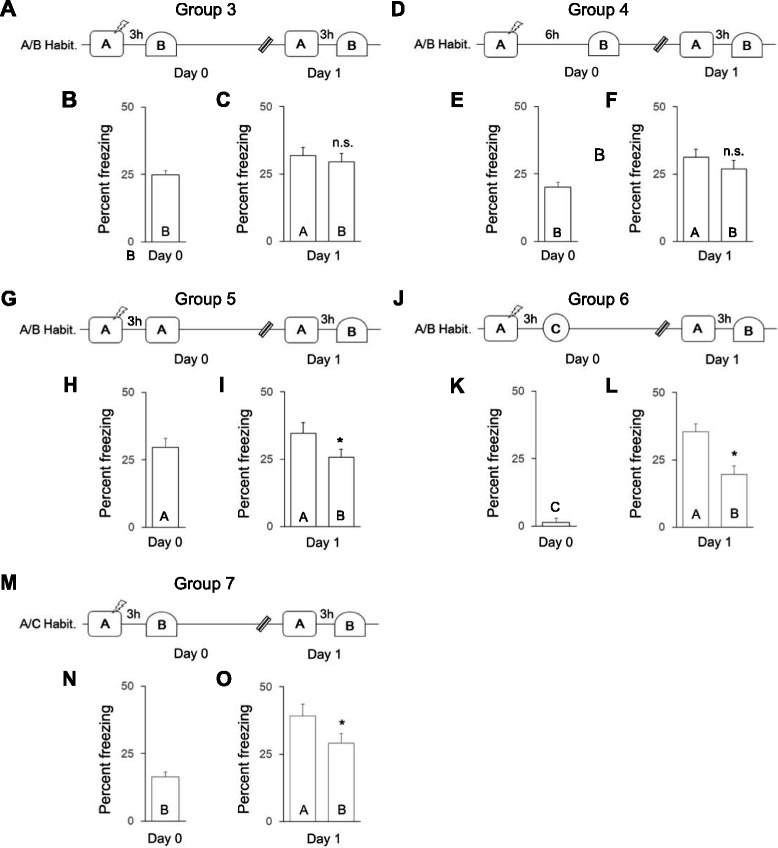
Fear generalization when mice were exposed to familiar contexts soon after training. **a**, **d**, **g**, **j**, **m** Experimental timelines for mice in Group 3, 4, 5, 6 and 7, respectively. Freezing in each context on (**b**, **e**, **h**, **k**, **n**) day 0 and (**c**, **f**, **i**, **l**, **o**) day 1 after training. A/B or A/C Habit. indicates habituation to contexts A and B or A and C before training, respectively. The double diagonal line in the timelines indicates a change in day, and the lightning bolt indicates the foot shock. Post-hoc analyses results: *p < 0.05, n.s. = not significant

We further examined which aspects of context exposure induce memory generalization. We exposed mice in Group 3 (Fig. 2a, *n* = 12) to the familiar, unconditioned context B 3 h after training and examined whether generalization occurred on day 1. The level of freezing in context B on day 0 was similar between mice in Group 3 and mice in Group 2 (compare Figs. 2b and 1e, independent samples *t*-test, *t*(40) = 1.9, *p* > 0.05), suggesting that exposure to context A on day 0 did not affect the freezing response to context B on day 0. Also, mice in Group 3 exhibited comparable levels of freezing in contexts A and B on day 1 (Fig. 2c, paired *t*-test, *t*(11) = 0.70, *p* > 0.05), similar to that observed for mice in Group 2 on day 1 (compare Fig. 2c and 1f, group × context ANOVA, group × context interaction, *F*(1,40) = 0.4, *p* > 0.05), indicating that exposure to a familiar context 3 h after training results in generalization. Furthermore, when the order of context exposure on day 1 was reversed for mice in Group 9 (i.e., context B before context A; Additional file 1: Figure S3A), generalization was still observed when context exposure occurred soon after training (Additional file 1: Figure S3C, A_day1_ ≅ B_day1_, paired *t*-test, *t*(11) = 0.6, *p* > 0.05), similar to that observed for mice in Group 3 (compare Additional file 1: Figure S3C and 2C, group × context ANOVA, group × context interaction, *F*(1,22) = 0.01, *p* > 0.05), indicating that memory generalization is unaffected by testing order.

Mice in Group 4 (Fig. 2d, *n* = 12) were exposed to context B 6 h after training (i.e., the same delay as in Group 2). Again, mice exhibited comparable levels of freezing in contexts A and B on day 1 (paired *t*-test, *t*(11) = 1.1, *p* > 0.05), indicating that exposure to a familiar context 6 h after training also results in generalization.

Generalization did not occur when the conditioned and unconditioned contexts were highly dissimilar (Additional file 1: Figure S1, contexts A and C; Additional file 1: Figure S4, Group 10; Additional file 1: Figure S4C, A_day1_ > C_day1_, paired *t-*test, *t*(12) = 6.8, *p* < 0.01). However, generalization still occurred when habituation time was reduced (Additional file 1: Figure S5, Group 11; Additional file 1: Figure S5C, A_day1_ ≅ B_day1_, paired *t*-test, *t*(11) = 1.4, *p* > 0.05), when habituation occurred 1 week before training (Additional file 1: Figure S6, Group 12; Additional file 1: Figure S6C, A_day1_ ≅ B_day1_, paired *t*-test, *t*(10) = 0.5, *p* > 0.05), and when habituation occurred only in the unconditioned context (Additional file 1: Figure S7, Group 13; Additional file 1: Figure S7C, A_day1_ ≅ B_day1_, paired *t*-test, *t*(10) = 1.4, *p* > 0.05). Together, these results suggest that exposure to a familiar, unconditioned context soon after learning (i.e., within 6 h) induces memory generalization.

Next, we examined whether exposure to the conditioned context 3 h after training induces generalization in mice in Group 5 (Fig. 2g, *n* = 12). Although mice in Group 5 showed more freezing in context A than in context B on day 1 (Fig. 2i, paired *t*-test, *t*(11) = 1.9, *p* < 0.05), the level of discrimination on day 1 was similar to that of Group 3 (compare Fig. 2i and c, group × context ANOVA, group × context interaction, *F*(1,40) = 0.4, *p* > 0.05), suggesting that exposure to a pre-habituated conditioned context soon after learning induces modest generalization.

Furthermore, we examined whether exposure to a novel context 3 h after training induces generalization. Mice in Group 6 (Fig. 2j, *n* = 10) were habituated to contexts A and B and then exposed to a novel and highly dissimilar context C after training. These mice exhibited a low level of freezing in context C on day 0 (Fig. 2 k) compared to mice in context B, Group 3 on day 0 (compare Fig. 2b and k, independent samples *t*-test, *t*(20) = 9.5, p < 0.01). They also showed greater freezing in context A than in context B on day 1 (Fig. 2l_,_ paired *t*-test, *t*(9) = 3.4, *p* < 0.005), in contrast to mice in Group 3 on day 1 (compare Fig. 2l and c, group × context ANOVA, group × context interaction, *F*(1,20) = 5.6, *p* < 0.05), suggesting that exposure to a novel, dissimilar context soon after training does not induce generalization. Mice in Group 7 (Fig. 2m, *n* = 12) were habituated to contexts A to C and then exposed to a novel but similar context B after training. These mice showed less freezing in context B on day 0 compared with mice in Group 3 (compare Fig. 2n and b, independent samples *t-*test, *t*(22) = 1.8, *p* < 0.05), indicating a reduced freezing response to an unfamiliar context after training. However, although mice in Group 7 exhibited greater freezing in context A than in context B on day 1 (Fig. 2o, paired *t-*test, *t*(11) = 2.0, *p* < 0.05), the level of discrimination on day 1 was similar between mice in Group 7 and mice in Group 3 (compare Fig. 2o and c, group × context ANOVA, group × context interaction, *F*(1,22) = 1.6, *p* > 0.05), suggesting that exposure to a novel but similar context soon after learning induces modest generalization.

Finally, as further support for our findings that exposure to a familiar context soon after learning induces generalization, we calculated discrimination indices for mice in Groups 1–7 (Additional file 1: Figure S8) [6, 8]. We found that mice in Groups 1 and 6 showed equivalently high discrimination indices (post-hoc analyses, independent-samples *t-*test, *t*(23) = 1.3, *p* > 0.05), whereas all other groups of mice showed lower discrimination indices than mice in Group 1 (group ANOVA, main effect of group, *F*(6,96) = 4.0, *p* < 0.01; post-hoc analyses, independent-samples *t-*tests, *ps* < 0.05).

## Discussion

We identified two important factors influencing whether fear memory generalizes to a non-fearful context. We found that both the timing of context exposure after fear learning and the familiarity of those contexts affect whether fear memory generalizes to a neutral context in which no aversive events happened. Specifically, our findings suggest that exposure to a familiar, unconditioned context soon after learning (i.e., within 6 h) but not long after learning (i.e., 24 h) induces memory generalization.

Recently, a considerable amount of evidence suggests that memory retrieval can trigger a second wave of consolidation processes that can either render the original memory liable to disruption [20] or strengthen the original memory [21]. This process is called memory reconsolidation [22, 23]. From this perspective, one could consider that the generalization observed in Group 2 was due to exposure to a neutral context (i.e., context B) during the time window when the memory of a fearful context (i.e., context A) was being retrieved and reconsolidated (i.e., after re-exposure to context A after training). However, retrieval of a fearful memory does not appear to be a necessary condition for generalization. That is, mice in Groups 3 and 4 exhibited generalization even though they were not re-exposed to context A after training. Also, mice in Group 1 did not show generalization even though they were exposed to context A after training. Therefore, it is more likely that exposure to a familiar but neutral context during the time window of initial fear memory consolidation (Group 3 and 4) or an interaction between initial consolidation and reconsolidation soon after learning (Group 2) leads to fear memory generalization.

After fear conditioning in one context, a fearful response to a second context could occur through two possible routes. First, a fearful or threatening event could also occur in the second context that was initially neutral. Second, no explicit threats may occur in the second context, but a fear memory could generalize from the first context. For the first route, the formation of a contextual fear memory initially requires activation of N-methyl-D-aspartate (NMDA) receptors [24, 25], but the formation of a second fear memory in a different context is insensitive [26] or less sensitive to the inhibition of NMDA receptors in mice [27] and rats [28]. Therefore, it would be interesting to know whether the second route also requires activation of NMDA receptors.

Generally, longer intervals between learning and treatments delivered after learning render those treatments less effective in altering memories [29, 30], which appears to hold true for the fear memory generalization observed in the present study. This time-limited effectiveness of treatments that alter fear memory is also found in ‘behavioral tagging’, which occurs when weak training produces long-term memories when paired with an arbitrary behavioral event that induces protein synthesis [31–33]. However, there are at least two differences between behavioral tagging and memory generalization. First, behavioral tagging addresses how a memory is formed and later expressed within a single context, whereas memory generalization addresses how a memory that was originally formed in one context is later expressed in a second context. Second, the novelty of the intervention is necessary for behavioral tagging [32], whereas it is not essential for memory generalization, as found in the present study. However, it would be interesting for future studies to investigate whether these phenomena share overlapping mechanisms.

## Conclusions

Mice exhibit generalized fear responses after exposure to a familiar, unconditioned context soon after a fearful experience. These findings increase our understanding of the conditions under which fear memory generalization occurs.

## Methods

### Animals

Mice (C57BL/6; Jackson Laboratory) were bred in our colony at the University of Tsukuba and maintained on a 12-h light/dark cycle (lights on 9 am-9 pm) with ad libitum access to food and water. All mice were group-housed at 2–4 mice/cage, and only male mice were used in the experiments. Before behavioral experiments, all mice were handled for 2 min twice per day for 5 days. All experiments were performed during the light cycle and conducted in accordance with the Science Council of Japan’s Guidelines for Proper Conduct of Animal Experiments. Experimental protocols were approved by the Animal Care and Use Committee at the University of Tsukuba.

### CFC

The contexts used in the CFC experiments were as previously described [8] with some modifications (Additional file 1: Figure S1). Context A consisted of a stainless steel conditioning chamber (31 × 24 × 21 cm; MED Associates) containing a stainless steel grid floor. The grid floor was composed of bars (3.2 mm diameter) spaced 7.9 mm apart that allowed the delivery of electric shocks. Underneath the grid floor was a stainless steel drop pan, which was lightly cleaned with 75 % ethanol that also provided a background odor. The front, top, and back of the chamber were made of clear acrylic, and the two sides were made of aluminum panels. Context B was similar to context A except that the floor and sides of the chamber were covered in white plastic sheets, and a piece of cardboard with a blue and white rectangular pattern was affixed to the front wall. Context C consisted of a circular glass chamber (22 cm diameter) and a cardboard floor covered with the same bedding material as used in the home cages. Ethanol odor was not used in contexts B and C.

All mice received 3 consecutive days of habituation to allow familiarization to contexts A, B, or C prior to CFC (except for mice in Group 8, which did not receive habituation). During each habituation session, mice were individually placed in a context for 5 min (except for mice in Group 11, which were placed in a context for 1 min) and then returned to their home cages. Habituation consisted of two sessions per day (except for mice in Group 13, which received one session per day). The inter-session interval within days was approximately 3 h. The sequence of habituation sessions was pseudorandomized (e.g., for A/B habituation, mice were exposed to context B and then context A on day −3, context A and then context B on day −2, and context B and then context A on day −1). For some mice (Groups 7 and 10), context C was used in place of context B, but other experimental parameters (i.e., timing, exposure duration, etc.) were the same.

One day after habituation (day 0; except for mice in Group 12, which were trained 1 week after habituation), mice underwent a training session, which consisted of receiving one electrical foot shock (0.5 mA, 2 s) 3 min after being placed in context A. Mice were returned to their home cages 2 min after the shock. After training, mice underwent multiple testing sessions at various time points, during which they were exposed to contexts A, B, or C for 3 min. The presence of freezing in the testing contexts was used as a behavioral index of a context-fear association [34]. Freezing behavior was measured using an automated scoring system (Actimetrics), which digitized the video signal at 4 Hz and compared frame-by-frame changes in mouse position.

Freezing data were subject to statistical analysis. Analysis of variance (ANOVA) or paired *t*-tests were used for within-group comparisons to determine the conditioning effect (e.g., freezing in context A before vs. after conditioning) and presence of context discrimination (e.g., freezing in context A vs. context B or C). Shapiro-Wilk tests were performed using SPSS software (IBM) to ensure that freezing data did not violate the assumption of normality for paired *t*-tests. Mixed-design ANOVA or independent samples *t*-tests were used for between-group comparisons. For mice in Groups 1, 2, and 8, *t*-tests were accompanied by repeated measures ANOVA. Type I error was set at 0.05. Data are shown as mean ± standard error.

## Additional file

Additional file 1: Figure S1. Front view of contexts A, B, and C. Pictures of contexts A (A), B (B), and C (C). Context A is similar to context B but dissimilar to context C. **Figure S2.** No fear generalization without habituation sessions. (A) Experimental timeline for mice in Group 8 (*n* = 10). Freezing in each context on (B) day 0 and (C) day 1. The double diagonal line in the timeline indicates a change in day, and the lightning bolt indicates the foot shock. Post-hoc analysis result: **p* < 0.05. **Figure S3.** Fear generalization without dependency on test order. (A, D) Experimental timelines for mice in Group 9 (*n* = 12). Freezing in each context on (B) day 0 and (C) day 1 after training. A/B Habit. indicates habituation to contexts A and B before training. The double diagonal line in the timelines indicates a change in day, and the lightning bolt indicates the foot shock. Post-hoc analysis result: n.s. = not significant. **Figure S4.** Fear discrimination after exposure to a familiar but highly dissimilar context. (A) Experimental timeline for mice in Group 10 (*n* = 13). Freezing in each context on (B) day 0 and (C) day 1 after training. A/C Habit. indicates habituation to A and C before training. The double diagonal line in the timeline indicates a change in day, and the lightning bolt indicates the foot shock. Post-hoc analysis result: **p* < 0.01. **Figure S5.** Fear generalization with shorter habituation sessions. (A) Experimental timelines for mice in Group 11 (*n* = 12). Freezing in each context on (B) day 0 and (C) day 1 after training. A/B Habit. indicates habituation to contexts A and B before training. The double diagonal line in the timeline indicates a change in day, and the lightning bolt indicates the foot shock. Post-hoc analysis result: n.s. = not significant. **Figure S6.** Fear generalization with habituation sessions 1 week before training. (A) Experimental timeline for mice in Group 12 (*n* = 11). Freezing in each context on (B) day 0 and (C) day 1 after training. A/B Habit. indicates habituation to contexts A and B before training. The double diagonal line in the timeline indicates a change in day, and the lightning bolt indicates the foot shock. Post-hoc analysis result: n.s. = not significant. **Figure S7.** Fear generalization with habituation only to context B. (A) Experimental timeline for mice in Group 13 (*n* = 11). Freezing in each context on (B) day 0 and (C) day 1 after training. B Habit. indicates habituation to context B before training. The double diagonal line in the timeline indicates a change in day, and the lightning bolt indicates the foot shock. Post-hoc analysis result: n.s. = not significant. **Figure S8.** Discrimination indices. Discrimination index was calculated as (freezing_contextA_ - freezing_contextB_)/max(freezing_contextA_, freezing_contextB_). The index is based on day 2 data for Group 1 and day 1 data for all other groups. Post-hoc analyses results: **p* < 0.05, n.s. = not significant. (PDF 1217 kb)

## Abbreviations

CFC: Contextual fear conditioning
NMDA: N-methyl-D-aspartate
