# Supplementary figures and images for: Effect of context exposure after fear learning on memory generalization in mice
Source: Mol Brain. 2016 Jan 8;9:2. doi: 10.1186/s13041-015-0184-0 (PMC4706703; doi:10.1186/s13041-015-0184-0)

**Figure S1**

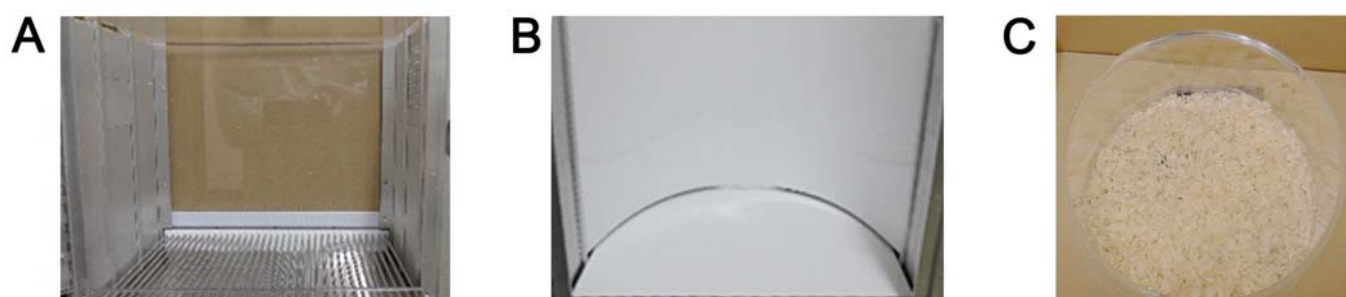

**Figure S2**

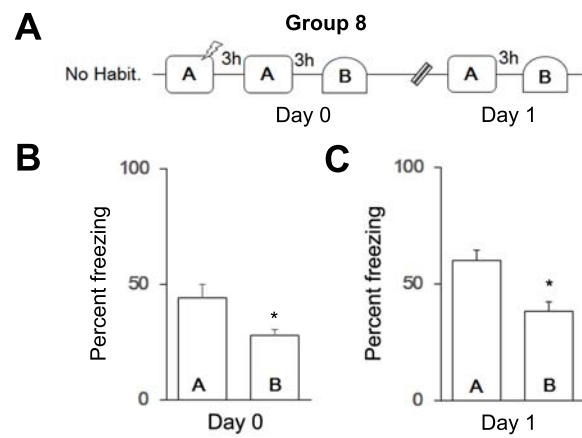

**Figure S3**

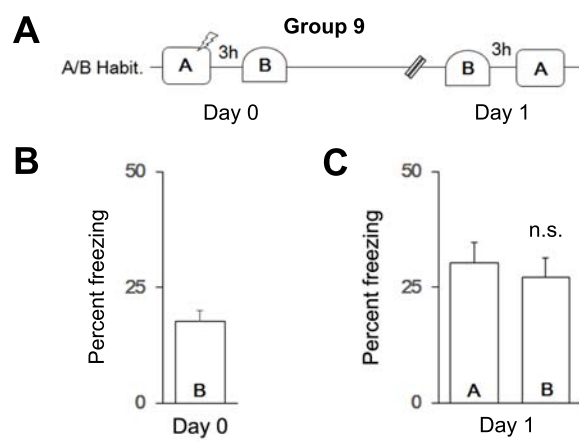

**Figure S4**

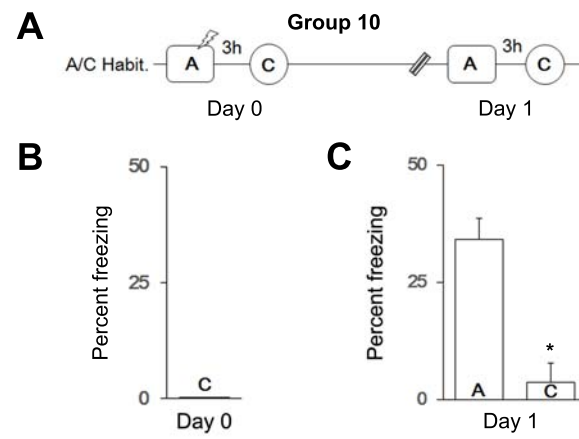

**Figure S5**

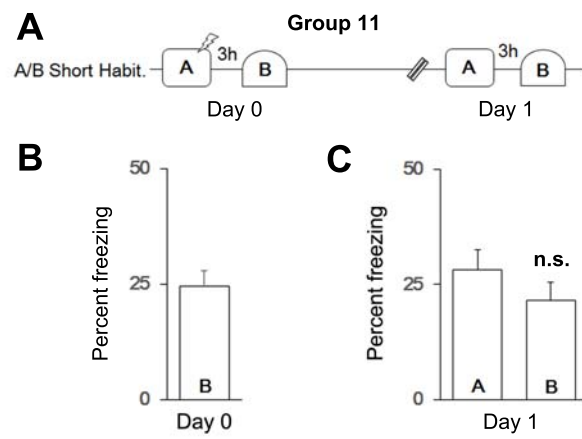

**Figure S6**

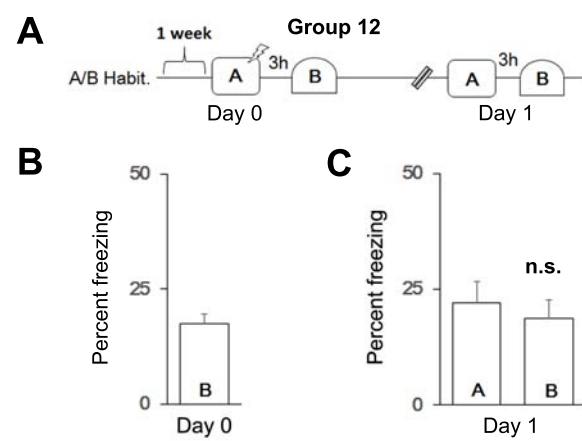

**Figure S7**

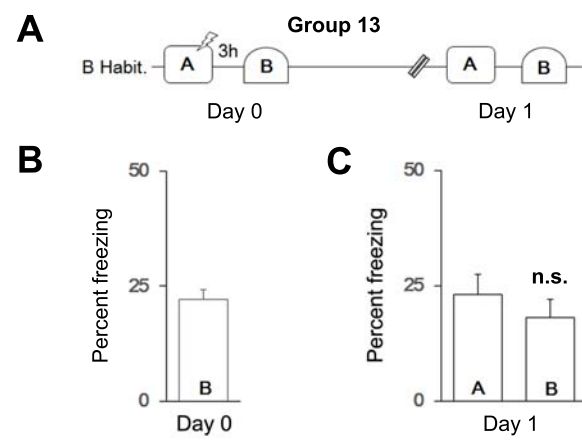

Figure S8

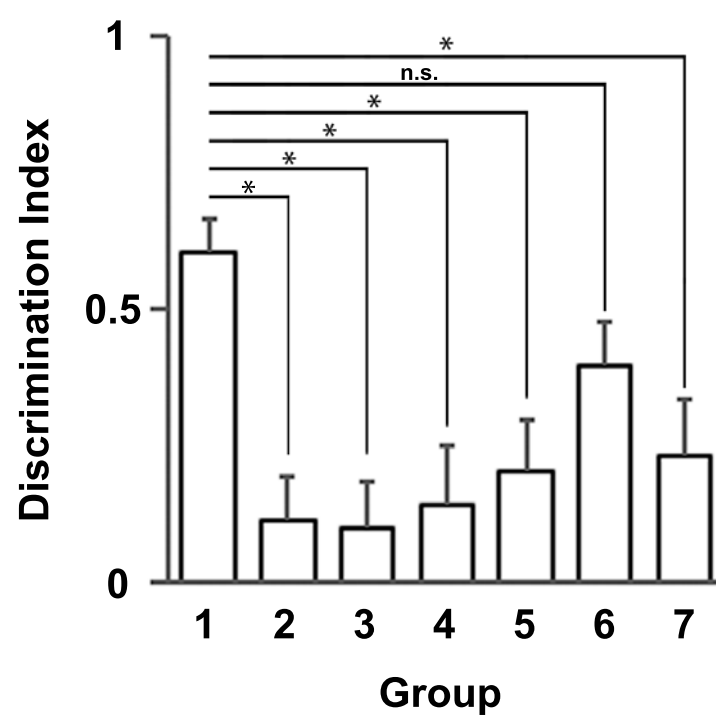

Supplement: Additional file 1: Figure S1. — Front view of contexts A, B, and C. Pictures of contexts A (A), B (B), and C (C). Context A is similar to context B but dissimilar to context C. Figure S2. No fear generalization without habituation sessions. (A) Experimental timeline for mice in Group 8 (n = 10). Freezing in each context on (B) day 0 and (C) day 1. The double diagonal line in the timeline indicates a change in day, and the lightning bolt indicates the foot shock. Post-hoc analysis result: *p < 0.05. Figure S3. Fear generalization without dependency on test order. (A, D) Experimental timelines for mice in Group 9 (n = 12). Freezing in each context on (B) day 0 and (C) day 1 after training. A/B Habit. indicates habituation to contexts A and B before training. The double diagonal line in the timelines indicates a change in day, and the lightning bolt indicates the foot shock. Post-hoc analysis result: n.s. = not significant. Figure S4. Fear discrimination after exposure to a familiar but highly dissimilar context. (A) Experimental timeline for mice in Group 10 (n = 13). Freezing in each context on (B) day 0 and (C) day 1 after training. A/C Habit. indicates habituation to A and C before training. The double diagonal line in the timeline indicates a change in day, and the lightning bolt indicates the foot shock. Post-hoc analysis result: *p < 0.01. Figure S5. Fear generalization with shorter habituation sessions. (A) Experimental timelines for mice in Group 11 (n = 12). Freezing in each context on (B) day 0 and (C) day 1 after training. A/B Habit. indicates habituation to contexts A and B before training. The double diagonal line in the timeline indicates a change in day, and the lightning bolt indicates the foot shock. Post-hoc analysis result: n.s. = not significant. Figure S6. Fear generalization with habituation sessions 1 week before training. (A) Experimental timeline for mice in Group 12 (n = 11). Freezing in each context on (B) day 0 and (C) day 1 after training. A/B Habit. indicates habituatio [file 13041_2015_184_MOESM1_ESM.pdf]
